# *Bacillus velezensis* A4 Against Fungal Pathogens via Membrane Integrity Disruption and Cellular Dysfunction in Fungal Pathogens

**DOI:** 10.3390/jof11120851

**Published:** 2025-11-29

**Authors:** Yu Xu, Hongyan Zhu, Wenxin Liu, Meng Wang, Xiaofeng Tang, Min Miao

**Affiliations:** 1 School of Food and Biological Engineering, Hefei University of Technology, Hefei 230601, China; 2 Engineering Research Center of Bio-Process, Ministry of Education, Hefei University of Technology, Hefei 230601, China

**Keywords:** *Bacillus velezensis*, cell-free supernatant, antifungal activity, omics analysis, cell membrane damage

## Abstract

Fruit decay caused by pathogenic fungi result in serious economic and quality losses during the postharvest stage. Biological control has evolved as a promising solution to these issues due to its environmentally friendly and safe characteristics. This study investigated the inhibitory effect of the biological control agent *Bacillus velezensis* A4 on various fungi and elucidated its antifungal mechanism. The strain demonstrated an antagonistic effect against 12 pathogenic fungi to different degrees, as well as the primary antifungal substances present in the cell-free supernatant (CFS). CFS induces the deformation of pathogenic hyphae and disrupts the permeability of hyphal cell membranes for effective biological control. Transcriptome and metabolome analysis showed that CFS disrupted lipid homeostasis and intracellular organization. In addition, the application of CFS leads to upregulation of membrane oxidation-related genes and the caspase gene family, thereby initiating the process of apoptosis. Our findings suggest that the broad-spectrum antifungal activity exhibited by *B. velezensis* A4 in CFS is due to the disruption of fungal cell membrane integrity and induction of fungal cell apoptosis-related pathways.

## 1. Introduction

Fruits are rich in high nutritional and water content, and exhibit increased metabolic activity, making them susceptible to postharvest pathogens, which can lead to yield and quality deterioration [1,2]. Fungal diseases are responsible for over 30% of losses during the storage and transportation stages of fruits [3]. Postharvest fruit decay is often caused by infection from specific fungi. For example, *Botrytis cinerea* can cause soft rot in strawberries and cherry tomatoes [4,5]; kiwifruit is susceptible to *Diaporthe nobilis* and *Botryosphaeria dothidea*; *Alternaria alternata* is an important pathogen for postharvest diseases of pomegranates [6]; and *Penicillium expansum* infection is commonly seen in apples, pears, and oranges [7]. While both chemical and biological approaches are employed to manage diseases, the growing concerns over pathogen resistance to fungicides and the adverse environmental and health impacts of chemical residues have promoted the search for safer alternatives for disease prevention and control [8,9].

Biological control agents utilize naturally antagonistic microorganisms to manage plant diseases in an eco-friendly and sustainable way [10,11]. *Bacillus* is one of the bacteria widely distributed in soil and rhizosphere as well as on the surface of plants, including *Bacillus subtilis*, *Bacillus velezensis*, *Bacillus cereus*, *Bacillus thuringiensis*, and *Bacillus amyloliquefaciens* [12,13,14,15,16], with a robust antagonistic ability against various pathogenic fungi on multiple fruits [17]. Therefore, *B. subtilis* is increasingly being used to control postharvest diseases [18]. The antifungal mechanisms include antibiotic production, nutrients and space competition, the secretion of extracellular hydrolytic enzymes, and the induction of plant defense responses [19,20,21,22]. A previous study showed that *B. subtilis* EA-CB0015 cells and their cell-free supernatant (CFS) suppressed the growth of nine fungal pathogens in vitro, primarily through secondary metabolites [23]. The CFS of *B. cereus* B8W8 had a significant effect on the inhibition of spores’ germination and mycelial growth of *P. expansum* and *B. cinerea* [24]. *B. velezensis* NH-13-5 could disrupt the integrity of fungal cell walls, thereby inhibiting the growth of *B. cinerea* in table grapes [25]. In addition, the CFS of *B. amyloliquefaciens* BA17 and *B. velezensis* A4 significantly restricted the mycelial growth of *B. cinerea*, indicating their potential as various fruit biocontrol agents [26,27]. Despite the extensive investigation, the molecular mechanisms of *Bacillus* against postharvest pathogens have not been fully explored.

In this study, *B. velezensis* A4 demonstrated broad-spectrum antifungal activity against 12 common plant pathogenic fungi, including *A. alternata*, *P. expansum*, *D. nobilis*, and *B. cinerea*, as evidenced by clear inhibition zones. The CFS of A4 induced morphological abnormalities of vesicles at the tips of pathogenic hyphae and altered organelle morphology. Further transcriptomic and metabolomic analysis revealed that CFS disrupted the fungal cell membranes and lipid metabolism. Additionally, the upregulation of membrane oxidation-related genes and caspase gene families suggests the activation of apoptosis-like pathways in fungal cells. These findings indicate that *B. velezensis* A4 exerts its antifungal effect by impairing cell integrity and triggering programmed cell death, thereby reducing the pathogenicity of fungi. Our findings indicate that *B. velezensis* A4 is a broad-spectrum biological control agent with potential applications in preventing postharvest diseases.

## 2. Materials and Methods

### 2.1. Identification of Pathogenic Fungi and Bacillus velezensis A4

The twelve pathogenic fungi used in the antifungal activity assay were initially isolated from diseased kiwifruit and identified through morphological characteristics, molecular analysis, and Koch’s postulates. Their ITS sequences were completely identical to the reference strains and have been deposited in GenBank with the following accession numbers: *Botryosphaeria dothidea* (MG282093.1), *Epicoccum nigrum* (MK656448.1), *Botrytis cinerea* (MG013937.1), *Alternaria alternata* (MG975630.1), *Colletotrichum gloeosporioides* (MN443603.1), *Didymella bellidis* (PQ219325.1), *Fusarium tricinctum* (MK594864.1), *Neofusicoccum mangiferae* (MG878158.1), *Neopestalotiopsis protearum* (MN635622.1), *Penicillium expansum* (NR_077154.1), *Pestalotiopsis trachicarpicola* (HM104328.1), *Diaporthe nobilis* (KT163359.1). All fungal strains were stored at −80 °C and cultured on potato dextrose agar (PDA) plates at 25 °C for 7–10 days prior to use [27].

*B. velezensis* A4 was identified and preserved by our laboratory. Its morphological characteristics, growth properties, and DNA analysis results were consistent with the established criteria for *B. velezensis*. Analysis of the 16S rRNA and *gyrB* genes (accession numbers MW828618 and MW900160) showed 99.93% and 99.43% sequence similarity, respectively, to the corresponding genes of the *B. velezensis* strain.

### 2.2. Cell-Free Supernatant Preparation from B. velezensis A4

The *B. velezensis* strain A4 was previously isolated and identified in our laboratory [27]. Glycerol bacteria stock was activated in a Luria–Bertani (LB) plate and incubated at 28 °C for 16 h. A single A4 colony was then inoculated into 2 mL of LB broth and cultured at 28 °C, with shaking at 180 rpm until the optical density at 600 nm (OD_600_) reached 0.6, which served as the seed culture. This seed culture was transferred (1% *v*/*v*) into 100 mL of fresh LB broth in a 250 mL conical flask and incubated at 28 °C for 72 h. After incubation, the bacterial culture was centrifuged at 10,000× *g* for 15 min at 4 °C. The resulting supernatant was carefully filtered through a 0.45 μm sterile membrane to obtain the cell-free supernatant (CFS).

### 2.3. Effectiveness of the CFS Against Diaporthe nobilis In Vivo

Mature kiwifruits (*Actinidia deliciosa* cv. Xuxiang) of uniform size and free of defects were surface-disinfected with 1% (*v*/*v*) sodium hypochlorite for 10 min, rinsed thoroughly, and air-dried [28]. A sterile needle was used to create uniform wounds (2 mm wide × 3 mm deep) along the equatorial region. For the CFS treatment, fruits were immersed in CFS solutions (0%, 5%, 10%, 15%) for 5 min and inoculated with 5 μL of *D. nobilis* spore suspension (1 × 10^6^ CFU/mL). For the fraction treatment, wounds were inoculated with the same spore suspension followed by application of 5 μL of sterile lipopeptide or protein fractions extracted according to Xiao et al. [10]; controls received an equal volume of ddH_2_O. All inoculation sites were sealed with parafilm, and fruits were incubated at 22–25 °C and 80–90% RH. Lesion diameters were measured after the indicated days, with eight fruits per treatment group.

### 2.4. Antifungal Experiment of B. velezensis A4

A dual-culture assay used to test the antagonistic activity of *B. velezensis* A4. Specifically, we placed a 5 mm test pathogen mycelial disk in the center of the PDA plate and marked two symmetrical inoculation points at 2 cm. A 5 µL aliquot of the A4 bacterial suspension (OD_600_ = 0.6) was spotted onto the point, while an equal volume of sterile LB medium was applied to the opposite point as a negative control. The inoculated plates were incubated at 25 °C. Antagonistic activity was assessed daily starting on day 3 by measuring the inhibition zone between the edge of the fungal colony and the bacterial inoculation spot. Each assay was performed with six biological replicates (*n* = 6).

### 2.5. Antifungal Assay of Volatile Components and CFS of A4 In Vitro

The antifungal effect of volatile organic compounds (VOCs) produced by strain A4 was evaluated using a divided Petri-dish assay [27]. Briefly, 15 µL of A4 bacterial suspension (6 × 10^8^ CFU/mL) was inoculated onto LB agar in one compartment of a divided plate, while an equal volume of sterile LB broth was applied to the corresponding compartment in the control group. The opposite compartment containing PDA, was inoculated with a fresh 5 mm mycelial plug of the pathogenic fungus. All plates were sealed with Parafilm to retain volatiles and incubated at 25 °C. Mycelial growth diameter and phenotypic changes were recorded at 4 and 6 days post-inoculation. Three independent experimental trials were conducted, each containing three biological replicates.

The inhibitory effect of A4 CFS on mycelial growth was assessed according to a previously described method [27] with modifications. Fresh 5 mm mycelial plugs of the pathogens were placed at the center of PDA plates supplemented with 2% (*v*/*v*) A4 CFS. Previous studies have confirmed that 2% is the minimum effective CFS concentration to inhibit the germination of *Botrytis cinerea* spores and mycelial growth [27]. Control plates contained pure PDA without CFS. All plates were incubated at 25 °C, and colony diameters were measured from the third day onward to calculate the mycelial growth inhibition rate. The experiment was conducted with six replicates per treatment.

### 2.6. Assessment of CFS Effects on Fungal Morphology

Fresh mycelial plugs (5 mm in diameter) of the test pathogen were inoculated into 30 mL of Potato Dextrose Broth (PDB) in conical flasks and pre-cultured at 25 °C with shaking at 120 rpm for 48 h. Subsequently, 600 µL of filter-sterilized CFS (2%) was added to the treatment flasks, while an equal volume of sterile water was added to the control flasks. After 12, 24, 36, and 48 h of further incubation under the same conditions, mycelial samples were collected at each respective time point, harvested by filtration, and washed twice with ice-cold phosphate buffer (0.05 M, pH 7.2) [27,28]. The hyphal morphology was then examined and documented using an optical microscope.

### 2.7. RNA Sequencing and Bioinformatic Analysis

Mycelia of *D. nobilis* treated with 2% CFS for 12 h were collected as described in Section 2.6, then immediately frozen in liquid nitrogen, and stored at −80 °C. Total RNA was isolated from individual samples (control: CK-1, CK-2, CK-3; treatment: T-1, T-2, T-3) and sequenced on the Illumina HiSeq platform (Illumina, San Diego, CA, USA). Qualified clean reads were aligned to the *D. nobilis* reference genome (ASM2307857v1) using HISAT2 (http://ccb.jhu.edu/software/hisat2/index.shtml, accessed on 29 October 2025). The gene differential expression analysis was performed using DESeq2 (http://bioconductor.org/packages/stats/bioc/DESeq2/, accessed on 29 October 2025), with a threshold of |log_2_ (fold change)| ≥ 1 and a false discovery rate (FDR) < 0.05 to identify significantly differentially expressed genes (DEGs). Functional enrichment analysis of the DEGs was conducted for Gene Ontology (GO) terms and Kyoto Encyclopedia of Genes and Genomes (KEGG) pathways using Goatools (https://github.com/tanghaibao/GOatools, accessed on 29 October 2025) and Python scipy software (https://scipy.org/install/, accessed on 29 October 2025), respectively, with a significant cutoff of adjusted * *p*-value (Padj) < 0.05.

### 2.8. Metabolomic Sample Preparation and Analysis

Mycelial samples of *D. nobilis* (50 mg) were collected as described in Section 2.6. Each sample was homogenized in 400 µL of pre-cooled 80% methanol aqueous solution containing 0.02 mg/mL L-2-chlorophenylalanine (internal standard). The mixture was ground and subjected to ultrasonication at 4 °C for 30 min. After centrifugation, the filter supernatants were analyzed using a Thermo UHPLC-Q Exactive system equipped (Thermo Fisher Scientific, Waltham, MA, USA) with an ACQUITY HSS T3 column (100 mm × 2.1 mm, 1.8 μm; Waters (Fisher Chemical, Waltham, MA, USA)). Progenesis QI software (v3.0, WatersCorporation, MA, USA) was used for the analysis and processing of raw LC-MS data. Metabolite identification was performed by comparing with the Human Metabolome Database (HMDB), Metlin, and Majorbio databases. ANOVA, PCA, and OPLS-DA analyses were conducted with seven-cycle cross-validation. Metabolites with Variable Importance (VIP) > 1 and *p* < 0.01 were classified as significantly different metabolites, and functional enrichment analysis was performed based on the KEGG database (https://www.genome.jp/kegg/, accessed on 29 October 2025).

### 2.9. Ultrastructural Analysis of Fungal Organelles

The procedure for transmission electron microscopy sample preparation followed the method described by Zeng et al. [29], with minor modifications. Briefly, fungal hyphae collected as described in Section 2.6 were fixed in 2.5% (*v*/*v*) glutaraldehyde at 4 °C for 0.5 h. After being rinsed three times with phosphate buffer (0.1 M, pH 7.2), the samples were post-fixed with 1% osmium tetroxide for 2 h at room temperature. Dehydration was performed using a graded ethanol series, followed by embedding in epoxy resin. Ultrathin sections (70–90 nm) were prepared and examined using a Hitachi HT7800 transmission electron microscope (HITACHI, Tokyo, Japan).

### 2.10. Quantitative Real-Time PCR

Total RNA was extracted from the RNA-seq mycelium sample utilizing Trizol reagent (Tiangen, Beijing, China), and cDNA was obtained using the cDNA synthesis kit (Vazyme, R323-01, Nanjing, China). The relative gene expression was analyzed by qRT-PCR using the HiscriptII 1st Strand cDNA Synthesis Kit (Vazyme, R323-01, Nanjing, China). All primer sequences of genes associated with oxidative membrane damage and caspase-related were listed in Supplementary Table S1. Gene expression levels were normalized to the actin gene and calculated using the 2^−∆∆Ct^ method. Each sample was analyzed in three biological replicates, with two technical repeats per experiment.

### 2.11. Statistical Analysis

All data were statistically analyzed using SPSS 23.0 and visualized with GraphPad Prism 8.0. One-way analysis of variance (ANOVA) followed by Duncan’s multiple range test was applied for multiple comparisons. The results are expressed as mean ± standard error of the mean (SEM). Differences at *p* < 0.05 were considered statistically significant, with *p* < 0.01 and *p* < 0.001 denoted as ** and ***, respectively.

## 3. Results

### 3.1. Antifungal Effect of Bacillus velezensis A4 Against Multiple Plant Pathogenic Fungi

**Figure 1:**
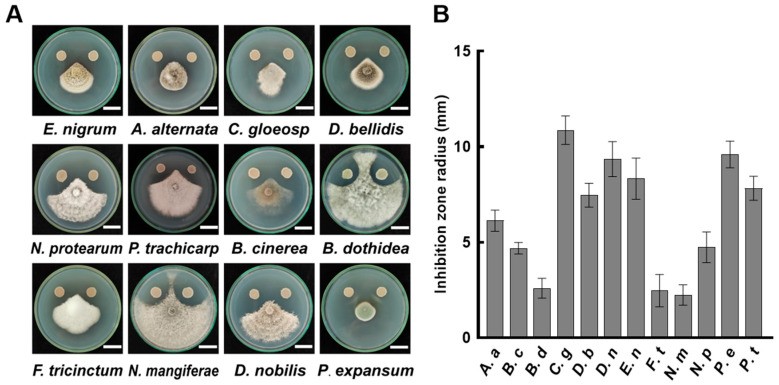
Antifungal activity of *Bacillus velezensis* A4 against plant pathogenic fungi. (**A**) Representative images of dual-culture inhibition assays between strain A4 and pathogenic fungi after 6 days of incubation. Scale bar = 2 cm. (**B**) Quantitative analysis of inhibition zone diameters (*n* = 6). *Epicoccum nigrum* (*E. nigrum*, *E. n*), *Alternaria alternata* (*A. alternata*, *A. a*), *Colletotrichum gloeosporioides* (*C. gloeosporioides*, *C. g*), *Didymella bellidis* (*D. bellidis*, *D. b*), *Neopestalotiopsis protearum* (*N. protearum*, *N. p*), *Pestalotiopsis trachicarpicola* (*P. trachicarpicola*, *P. t*), *Botrytis cinerea* (*B. cinerea*, *B. c*), *Botryosphaeria dothidea* (*B. dothidea*, *B. d*), *Fusarium tricinctum* (*F. tricinctum*, *F. t*), *Neofusicoccum mangiferae* (*N. mangiferae*, *N. m*), *Diaporthe nobilis* (*D. nobilis*, *D. n*), *Penicillium expansum* (*P. expansum*, *P. e*).

Pathogenic fungi are the primary cause of fruit decay. The results of the dual culture experiment showed that *B. velezensis* A4 exhibited significant antagonistic activity against 12 tested fungi (Figure 1). The representative results of inhibition on the sixth day of cultivation are shown in Figure 1A. Quantitative measurements revealed that *B. velezensis* A4 has an inhibition zone range of 2.24 mm to 10.87 mm across the tested fungi (Figure 1B). Among them, the strongest inhibition was observed on *Colletotrichum gloeosporioides* (10.87 mm), followed by *Penicillium expansum* (9.6 mm), *Diaporthe nobilis* (9.35 mm), and *Epicoccum nigrum* (8.33 mm). Even the least affected species, *Neofusicoccum mangiferae*, showed a measurable inhibition zone of 2.24 mm (Figure 1B). These results demonstrate that *B. velezensis* A4 exhibits broad-spectrum antifungal activity against a range of postharvest pathogenic fungi.

### 3.2. Inhibitory Effects of Volatile Components from B. velezensis A4

**Figure 2:**
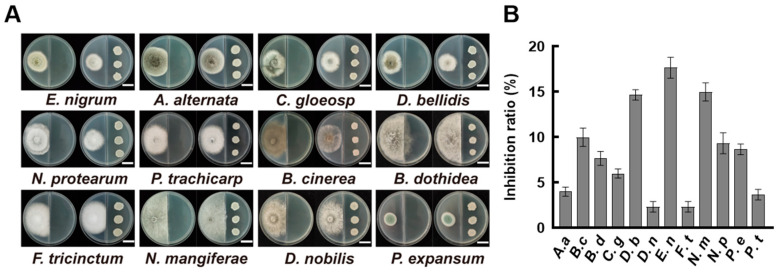
Inhibitory effects of volatile compounds from *Bacillus velezensis* A4 on pathogenic fungi. (**A**) Divided-plate assay evaluating fungal growth inhibition by A4-derived volatiles after 4 days of incubation. Scale bar = 2 cm. (**B**) Quantitative assessment of fungal colony diameters at day 4 (*n* = 6). *Epicoccum nigrum* (*E. nigrum*, *E. n*), *Alternaria alternata* (*A. alternata*, *A. a*), *Colletotrichum gloeosporioides* (*C. gloeosporioides*, *C. g*), *Didymella bellidis* (*D. bellidis*, *D. b*), *Neopestalotiopsis protearum* (*N. protearum*, *N. p*), *Pestalotiopsis trachicarpicola* (*P. trachicarpicola*, *P. t*), *Botrytis cinerea* (*B. cinerea*, *B. c*), *Botryosphaeria dothidea* (*B. dothidea*, *B. d*), *Fusarium tricinctum* (*F. tricinctum*, *F. t*), *Neofusicoccum mangiferae* (*N. mangiferae*, *N. m*), *Diaporthe nobilis* (*D. nobilis*, *D. n*), *Penicillium expansum* (*P. expansum*, *P. e*).

In order to assess the antifungal activity of volatile metabolites produced by *B. velezensis* A4, we employed a divided-plate assay against 12 plant pathogenic fungi. After four days of incubation, the volatiles released by strain A4 exhibited discernible but variable inhibitory effects on mycelial growth across different fungal species (Figure 2A). The inhibition rates were calculated based on the colony diameter. The results showed that *E. nigrum* received the highest inhibition rate at 17.67%, followed by *N. mangiferae* (15.00%). *Pestalotiopsis trachicarpicola*, *D. nobilis*, and *Fusarium tricinctum* showed slight minimal inhibition, with inhibition rates of 3.67%, 2.33%, and 2.32%, respectively (Figure 2B). These results indicate that volatile compounds from *B. velezensis* A4 play a limited role in fungal suppression and are not the strain’s primary mode of antagonism.

### 3.3. Cell-Free Supernatant of A4 Retards the Growth of Pathogenic Fungi

**Figure 3:**
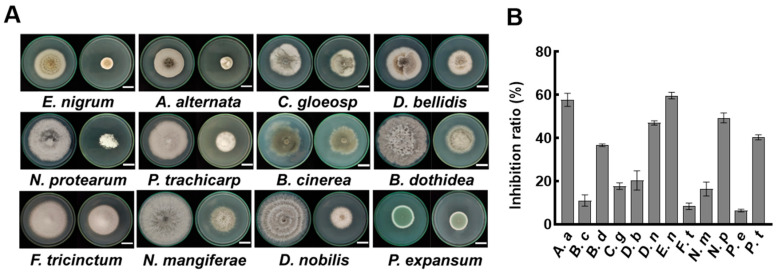
Effect of A4 cell-free supernatant (CFS) on pathogenic fungi. (**A**) Antifungal activity assay on PDA plates. Left panel shows control groups without CFS, and right panel displays treatment groups supplemented with 2% CFS. The images were recorded after 6 days of incubation. Scale bar = 2 cm. (**B**) Statistical analysis of fungal colony diameters at day 6 post-inoculation (*n* = 6). *Epicoccum nigrum* (*E. nigrum*, *E. n*), *Alternaria alternata* (*A. alternata*, *A. a*), *Colletotrichum gloeosporioides* (*C. gloeosporioides*, *C. g*), *Didymella bellidis* (*D. bellidis*, *D. b*), *Neopestalotiopsis protearum* (*N. protearum*, *N. p*), *Pestalotiopsis trachicarpicola* (*P. trachicarpicola*, *P. t*), *Botrytis cinerea* (*B. cinerea*, *B. c*), *Botryosphaeria dothidea* (*B. dothidea*, *B. d*), *Fusarium tricinctum* (*F. tricinctum*, *F. t*), *Neofusicoccum mangiferae* (*N. mangiferae*, *N. m*), *Diaporthe nobilis* (*D. nobilis*, *D. n*), *Penicillium expansum* (*P. expansum*, *P. e*).

Previous studies indicate that the antimicrobial components of *Bacillus* species are primarily present in the cell-free supernatant (CFS). We were prompted to examine the inhibitory effects of the CFS from strain A4 on various plant pathogenic fungi. Fungi were cultured on PDA and PDA plates supplemented with 2% CFS. After six days of incubation, the mycelia in the PDA plate showed normal growth status (Figure 3A). In contrast, mycelia cultured in a plate containing 2% CFS exhibited marked growth inhibition (Figure 3A). Among the 12 tested fungi, five strains had inhibition rates exceeding 40%, with the strongest suppression observed against *E. nigrum* at 59.45% (Figure 3B). Notably, 2% CFS showed inhibition rates of 40.33%, 47.00%, and 8.33% against *P. trachicarpicola*, *D. nobilis*, and *F. tricinctum*, respectively, which were significantly higher than the inhibition rates of volatile compounds against these strains. These results confirm the dominant role of CFS compounds in the antifungal activity of *B. velezensis* A4.

### 3.4. Effect of CFS on Mycelial Morphology of Pathogenic Fungi

**Figure 4:**
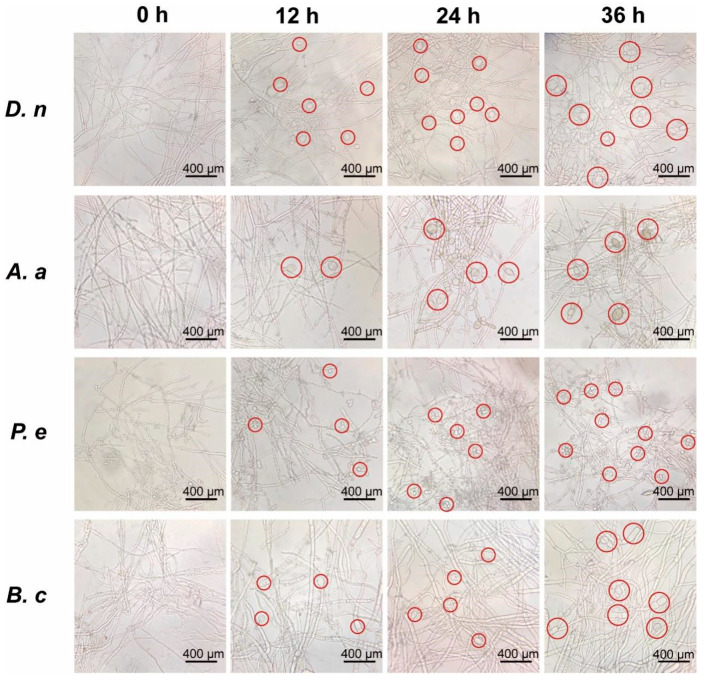
Morphological alterations of pathogenic fungi induced by 2% cell-free supernatant (CFS). Hyphal structures were observed by light microscopy after cultivation in potato dextrose broth supplemented with 2% CFS for indicated durations. Scale bar = 400 μm. Red circles representative morphological abnormalities. *Diaporthe nobilis* (*D. nobilis*, *D. n*), *Alternaria alternata* (*A. alternata*, *A. a*), *Penicillium expansum* (*P. expansum*, *P. e*), *Botrytis cinerea* (*B. cinerea*, *B. c*).

According to the finding that 2% CFS effectively retarded mycelial expansion on medium, we further investigated its morphological impact using liquid culture for more detailed observation. As shown in Figure 4, hypha of untreated pathogenic fungi (0 h) exhibited typical growth, characterized by thin-walled, transparent, and regularly branched hyphae forming a network structure. In contrast, exposure to 2% CFS induced increasing morphological aberrations over time across all 12 tested species. These alterations included swelling of apical hyphae, elongation of hyphal cells, and increased lateral branching (Figure 4 and Figure S1). The effect was particularly pronounced in *D. nobilis* and *Alternaria alternata*, where transparent vesicle-like structures appeared at hyphal tips after 12 h of treatment. By 36 h, severe deformation was evident, with individual cells appearing swollen and structurally compromised, indicating extensive CFS-induced damage.

### 3.5. CFS Alters the Ultrastructure of Pathogenic Fungi

**Figure 5:**
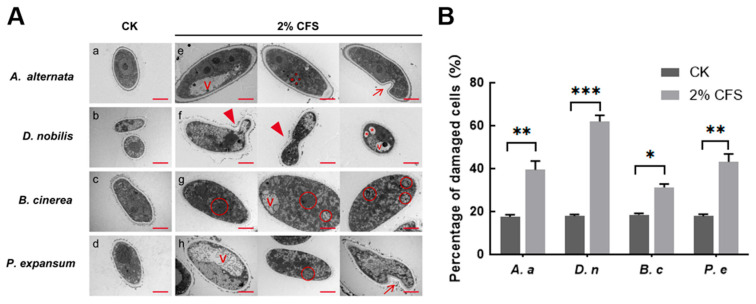
Effect of CFS treatment on pathogenic organelles. (**A**) (**a**–**d**) denote the control groups of *Alternaria alternata* (*A. alternata*, *A. a*), *Diaporthe nobilis* (*D. nobilis*, *D. n*), *Botrytis cinerea* (*B. cinerea*, *B. c*), and *Penicillium expansum* (*P. expansum*, *P. e*). (**e**–**h**) denote the treatment groups of *A. a*, *D. n*, *B. c*, and *P. e*, respectively. V (vesicles); → membrane invagination; ▲ budding vesicles; * nuclear disintegration fragmentation; red circle indicated autophagic bodies. Images represent three independent experiments. Magnification 12 K×. Scale bar = 2 μm. (**B**) Quantification of damaged cells. All data represent mean ± SEM. Asterisks indicate statistical significance (* *p* < 0.05, ** *p* < 0.01 and *** *p* < 0.001) compared with CK.

Observation under an optical microscope showed hyphal malformation caused by 2% CFS treatment (Figure 4), suggesting that the integrity of the cell membrane might have been compromised. We next observed the subcellular structure through transmission electron microscopy and found that the ultrastructure of fungal organelles was severely damaged after 24 h of treatment with 2% CFS. Untreated mycelial cells exhibit typical structure, including continuous cell walls, clear membrane structures, homogeneous cytoplasm, and well-structured organelles (Figure 5(Aa–Ad)). In contrast, mycelial samples treated with CFS generally exhibited structural disturbances (Figure 5(Ae–Ah)). Differential changes were observed in individual fungi after CFS treatment. *A. alternata* is characterized by cell swelling, membrane invagination, intracellular cavities, vesicle formation, and nuclear fragmentation (Figure 5(Ae)). The blurred nuclear membrane boundaries, intracellular vacuolization, and budding phenomena were observed in *D. nobilis* (Figure 5(Af)). The hyphae of *Botrytis cinerea* displayed changes in cytoplasmic density and accumulation of autophagosome-like structures (Figure 5(Ag)). *P. expansum* exhibited vacuolization, autophagosomes, and invaginated plasma membranes (Figure 5(Ah)). Quantitative analysis shows that 2% CFS leads to an increased rate of cell damage (Figure 5B). These observations indicate that CFS of A4 inhibits pathogenic hyphal growth mediated by disrupting membrane structure, genetic information, and intracellular homeostasis.

### 3.6. Suppressive Effect of CFS on D. nobilis Pathogenicity in Kiwifruit

**Figure 6:**
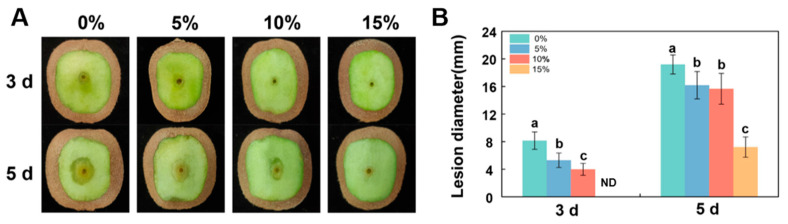
*Bacillus velezensis* A4 cell-free supernatant (CFS) reduced the pathogenicity of *Diaporthe nobilis* in kiwifruit. (**A**) Representative images of disease symptoms at 3- and 5-day post-inoculation. 5%, 10%, and 15% indicated that the CFS was diluted with distilled water to 5%, 10%, and 15% of the final working volume fraction. (**B**) Quantification of lesion diameters. Histograms with different letters indicate significant differences according to Duncan’s multiple range tests at *p* < 0.05. ND: Not Detected.

To evaluate the effect of *B. velezensis* A4 on the virulence of *D. nobilis* in vivo, we conducted fruit-inoculation experiments to confirm the biocontrol efficacy of its CFS. As shown in Figure 6, the control group (0%) developed lesions reaching 8.36 mm by day 3, whereas all CFS treatments significantly inhibited fungal expansion in fruit. The 5%, 10%, and 15% CFS treatments reduced lesion diameters in a dose-dependent manner, with the 15% CFS group showing complete suppression of symptom development on day 3. This dose-responsive inhibition pattern remained consistent after inoculation on day 5, suggesting its effective control of fruit postharvest decay in vivo.

### 3.7. Transcriptome Analysis of Differentially Expressed Genes

**Figure 7:**
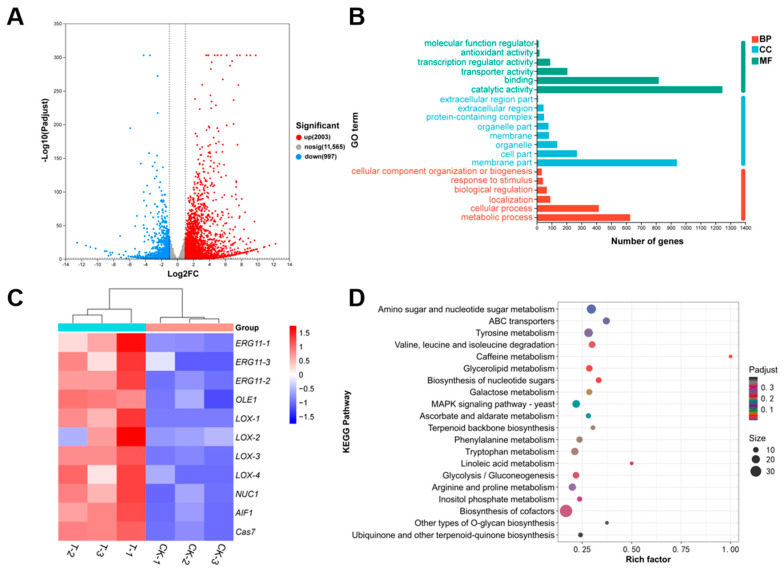
Transcriptomic analysis of *Diaporthe nobilis* under CFS treatment. (**A**) Volcano plot of differentially expressed genes identified from mycelium treated with 2% CFS for 12 h. Significantly down- and up-regulated genes are shown in blue and red, respectively. (**B**) Gene Ontology classification of differential genes across three major functional categories. BP: Biological Process; CC: Cellular Component; MF: Molecular Function. (**C**) Heatmap analysis of differentially expressed genes in *D. nobilis* under *Bacillus velezensis* A4 treatment, with color intensity indicating relative abundance changes. (control: CK-1, CK-2, CK-3; treatment: T-1, T-2, T-3). (**D**) KEGG pathway enrichment scatter plot displaying the top 20 significantly enriched pathways (Padj < 0.05). The vertica axis indicates pathway names, and the horizontal axis represents the rich factor. Point size corresponds to the number of enriched genes, and color intensity reflects the Padj value.

To elucidate the molecular mechanism of CFS-mediated inhibition of fungal growth, RNA-seq analysis was performed on *D. nobilis* mycelium treated with 2% CFS for 12 h (PRJPRJNA1357093). *D. nobilis* was further chosen as a representative strain for RNA-Seq analysis based on its established role as a major and widespread postharvest pathogen of kiwifruit [30,31] and sensitivity to CFS treatment (Figure 3, Figure 4 and Figure 5). Transcriptome analysis identified 3000 differentially expressed genes (DEGs) in the *D. nobilis* samples treated with CFS, comprising 2003 up-regulated genes and 997 down-regulated genes (Figure 7A). Gene Ontology (GO) enrichment analysis of substantial changes in cellular components revealed that membrane-related components were most significantly enriched (940 genes), followed by cell part (269 genes) and organelles (137 genes) (Figure 7B). These findings indicate that CFS mainly targets cell membranes and subcellular structures, consistent with the observation of changes in hyphal structures under the microscope. The heatmap of DEGs showed that numerous genes involved in lipid metabolism and apoptosis pathways were coordinately up-regulated in the CFS treatment group (Figure 7C). The ergosterol synthesis gene *ERG11* (*ERG11-1*, *ERG11-2*, *ERG11-3*), the fatty acid desaturase gene *OLE1*, and the lipoxygenase gene *LOX* (*LOX-1*/*2*/*3*/*4*), which are critical for lipid metabolism, showed significant induction after 12 h of treatment with 2% CFS. Moreover, the apoptosis-related genes, including mitochondrial nuclease *NUC1*, apoptosis-inducing factor *AIF1*, and cysteine aspartate protease gene *Cas7*, were also elevated upon CFS application. These results indicated a dual antifungal strategy of A4, which simultaneously targets membrane integrity and triggers apoptotic pathways.

Kyoto Encyclopedia of Genes and Genomes (KEGG) pathway analysis was performed to identify key metabolic pathways affected by CFS treatment in *D. nobilis* (Figure 7D). The three pathways with significant enrichment include amino acid and nucleotide sugar metabolism (24 genes), which participate in the core metabolic processes supporting cell growth and energy supply; ABC transporters (16 genes) promote transmembrane molecular transport and are associated with nutrient acquisition and detoxification; Tyrosine metabolism (22 genes) is involved in the biosynthesis, degradation, and regulation of cell development [32]. The treatment of CFS interferes with the biological functions of fungi by targeting multiple cellular processes, including essential metabolic activity, membrane transport homeostasis, and cellular developmental signaling networks.

### 3.8. Metabolome Differential Metabolite Analysis

**Figure 8:**
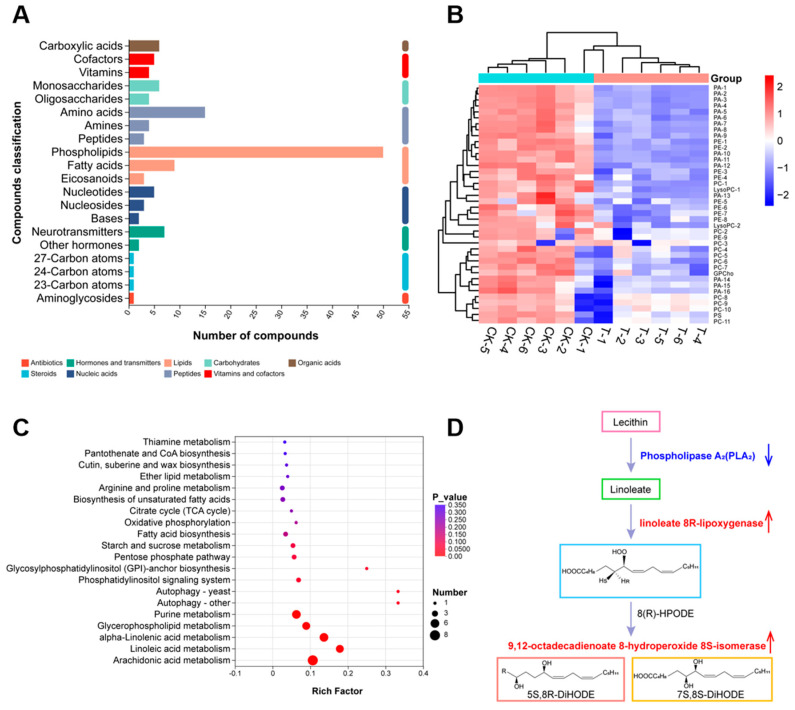
Metabolomic profiling of *Diaporthe nobilis* under CFS stress. (**A**) Statistical plot of differential metabolite classification. The vertical coordinate is the secondary classification category of compounds, and the horizontal coordinate is the number of metabolites annotated to that category. (**B**) Heatmap representation of phospholipid metabolites, with color intensity indicating relative abundance changes. (control: CK-1, CK-2, CK-3; treatment: T-1, T-2, T-3). (**C**) KEGG enrichment analysis of differential metabolites, displaying the top significantly altered pathways with bubble size representing the number of enriched metabolites. (**D**) Schematic representation of key enzymatic regulation in linoleic acid metabolism under *Bacillus velezensis* A4 treatment. Blue downward arrow denotes down-regulation of phospholipase A_2_, suppressing linoleic acid synthesis. Red upward indicate up-regulation of linoleate 8R-lipoxygenase and 9,12-octadecadienoate 8-hydroperoxide 8S-isomerase, enhancing degradation pathways.

To further characterize the functional consequences of the transcriptional changes, we performed non-targeted metabolomic analysis of *D. nobilis* under CFS stress (MTBLS13351). This analysis identified 131 differential metabolites, which were classified into nine categories including lipids (46.96%), peptides (16.79%), nucleic acids (7.62%), carbohydrates (7.62%), vitamins and cofactors (6.87%), hormones and transmitters (6.87%), organic acids (4.58%), steroids (2.29%), and antibiotics (0.76%) (Figure 8A). Among the lipids, 49 phospholipids (80.33%) were detected (Figure S2), with significant down-regulation of phosphatidic acid (PA, 16 species), phosphatidylethanolamine (PE, 9 species), phosphatidylcholine (PC, 11 species), and lysophosphatidylcholine (LysoPC, 2 species) (Figure 8B), all of which are key regulators of membrane fluidity and permeability. These results indicate that CFS disrupts the lipid homeostasis of the *D. nobilis* cell membrane. KEGG enrichment analysis revealed significant changes in lipid metabolism pathways, including alpha-linolenic acid, linoleic acid, arachidonic acid, glycerophospholipid, and glycerolipid (Figure 8C). The polyunsaturated fatty acids in these pathways are key components of membrane phospholipids. It is worth noting that the down-regulation of phospholipase A_2_ gene (encoding phospholipase A_2_, PLA_2_, EC 3.1.1.4) inhibits the synthesis of linoleic acid, while the up-regulation of linoleate 8R-lipoxygenase (encoding linoleate 8R-lipoxygenase EC 1.13.11.60) and 9,12-octadecadienoate 8-hydroperoxide 8S-isomerase (encoding 9,12-octadecadienoate 8-hydroperoxide 8S-isomerase EC 5.4.4.6) enhances their degradation (Figure 8D). These metabolic shifts reflect severe disruption of membrane integrity and stability in *D. nobilis* under CFS stress.

### 3.9. CFS Induces Expression of Membrane Oxidation and Apoptosis-Related Genes in D. nobilis

**Figure 9:**
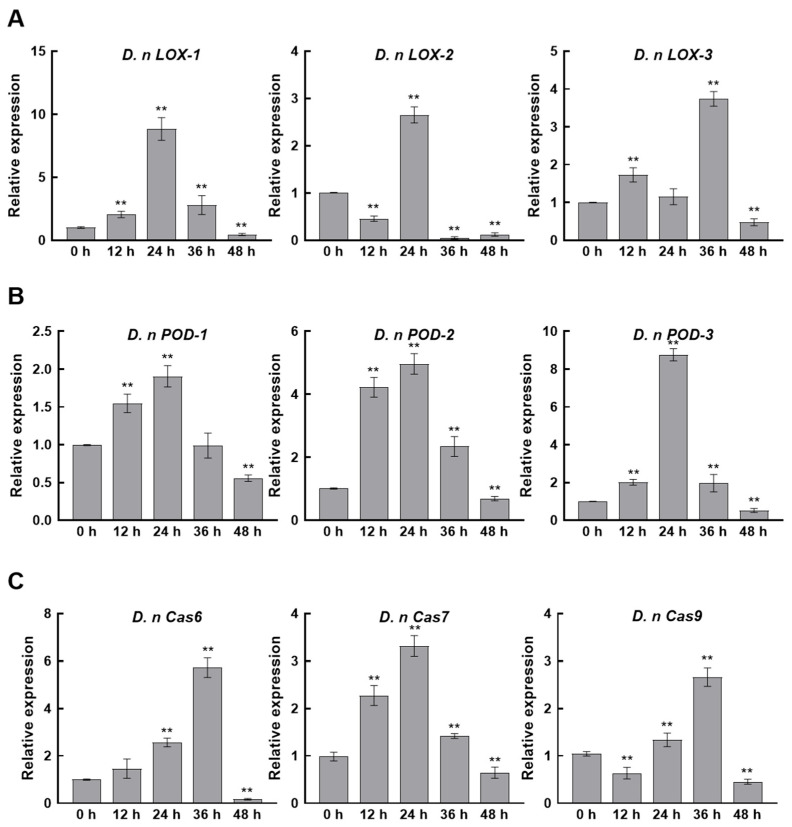
Expression levels of membrane oxidation- and apoptosis-related genes in *Diaporthe nobilis* under CFS treatment. (**A**) Relative expression levels of lipoxygenase (*LOX*) genes, (**B**) peroxidase (*POD*) genes, and (**C**) Caspase (*cas*) genes in response to CFS treatment. All data represent mean ± SEM. Asterisks indicate significant differences (** *p* < 0.01) as determined by Student’s *t*-test.

Transcriptomic and metabolomic analyses revealed a substantial disruption of lipid homeostasis in pathogenic fungi, accompanied by structural damage as observed by electron microscopy. We further detected the expression levels of membrane integrity and apoptosis-like processes through qRT-PCR. Specifically, the expression levels of lipoxygenase (*LOX*) and peroxidase (*POD*) genes, which are involved in membrane lipid peroxidation, and the caspase family genes associated with programmed cell death, were analyzed. After CFS treatment, the expression of *D. nLOX-1*, *D. nLOX-2*, and *D. nLOX-3* in *D. nobilis* was rapidly up-regulated within 12 to 24 h. Their peak expression levels increased by 8.77-, 2.55-, and 3.64-fold, respectively (Figure 9A). Parallel expression patterns were observed for three peroxidase genes, *D. nPOD-1*, *D. nPOD-2*, and *D. nPOD-3*, which reached maximum induction at 24 h with 1.84-, 4.76-, and 8.75-fold up-regulation, respectively (Figure 9B). These coordinated gene expression changes indicate CFS induced oxidative damage to the fungal cell membrane. Analysis of caspase gene expression revealed concurrent activation of apoptotic pathways in *D. nobilis*. The caspase genes *D. nCas6*, *D. nCas7*, and *D. nCas9* showed peak up-regulation of 5.62-, 3.13-, and 2.59-fold at 24 to 36 h post treatment (Figure 9C). These results demonstrate that CFS simultaneously triggers oxidative membrane damage and caspase-mediated apoptosis, collectively contributing to mycelial growth inhibition.

## 4. Discussion

The biocontrol bacteria exert their inhibitory effects on pathogens through various mechanisms such as producing antimicrobial compounds, competing for ecological niches with pathogens, and inducing host resistance [33]. Compared with synthetic fungicides, biocontrol agents have the advantages of high safety, minimal ecological impact, and low drug resistance [10,34]. Among numerous biocontrol agents, the *Bacillus* genus is widely applied for its broad-spectrum inhibitory activity against fungi, bacteria, and soilborne pathogens [33,35,36]. Although most strains of *Bacillus subtilis* exhibit antifungal properties against specific pathogens [37], only a few have shown the ability to inhibit multiple different fungal species simultaneously. The *B. subtilis* strain EA-CB0015 can inhibit nine different fungal pathogens [23]. In our previous work, we isolated *Bacillus velezensis* A4 from the surface of kiwifruit, demonstrating its ability to inhibit both mycelial growth and conidial germination of *Botrytis cinerea*, significantly reducing postharvest gray mold incidence [27]. The present study further establishes the broad-spectrum antifungal capacity of *B. velezensis* A4, which formed distinct inhibition zones against 12 plant pathogenic fungi (Figure 1). This pronounced inhibitory activity underscores the potential of *B. velezensis* A4 for postharvest disease management.

The volatile organic compounds (VOCs) of *B. subtilis* and the secondary metabolites in its cell-free supernatant (CFS) are the primary substances for its antimicrobial effects [24,38]. CFS of *B. subtilis* BS-1 induces hyphal expansion and rupture of *Botryosphaeria dothidea*, excessive accumulation of ROS, ultimately leading to cell death [28]. Previous studies found that *B. subtilis* CFS could exert antagonistic effects by inhibiting biofilm formation in *Pseudomonas* species [39]. Our research results showed that CFS of *B. velezensis* A4 exhibits broad-spectrum antifungal activity, significantly inhibiting the growth of all 12 tested pathogens, with particularly strong inhibitory effects on *Epicoccum nigrum* (59.45%) (Figure 3). It is noteworthy that the antifungal properties of VOCs and CFS vary substantially across different fungal strains. Specifically, while VOCs alone exhibited minimal inhibitory effects on certain fungi such as *Pestalotiopsis trachicarpicola* and *Diaporthe nobilis* (Figure 2), the application of 2% CFS led to a marked increase in inhibition rates (Figure 3), highlighting their complementary modes of action. This inhibitory difference suggests that a synergistic effect exists between the active ingredients in soluble metabolites and volatile components, and further research is necessary to identify the active compounds and assess their applications. Previous studies have confirmed that the primary antifungal compounds in *Bacillus* CFS are lipopeptides and protein fractions [36]. To this end, we isolated lipopeptides and protein fractions from the CFS of strain A4. As shown in Figure S3, both fractions significantly suppressed lesion expansion by *D. nobilis* in kiwifruit in vivo, confirming their functional role in the observed antifungal activity of CFS. The phenotype of cell swelling, vesicle formation, and abnormal hyphal branching observed after 36 h of treatment with 2% CFS indicates severe damage to membrane integrity (Figure 4). These findings are consistent with established research on *Bacillus*-derived metabolites that reduce membrane surface tension and enhance permeability [19,40]. Further subcellular observation of hyphae revealed that after 24 h of exposure to 2% CFS, pathogenic fungi exhibited ultrastructural changes. Compared with the intact cell structure observed in the control group (Figure 5(Aa–Ad)), the hyphae treated with CFS exhibited species-specific but convergent damage patterns (Figure 5(Ae–Ah)), mainly exhibiting the disruption of the cell membrane, organelles, and the nucleus. These results indicate that *B. velezensis* A4 could simultaneously target multiple cellular components, ultimately achieving irreversible inhibition of hyphal growth by disrupting cellular homeostasis.

*B*. *velezensis* A4 demonstrates distinctive antifungal mechanisms through integrated multi-omics analysis. The strain produces a unique metabolite profile characterized by atypical lipopeptide composition and several uncharacterized antifungal compounds (Figure 8), suggesting novel secondary metabolites that contribute to its broad-spectrum activity. qRT-PCR analysis revealed that the cell-free supernatant exerts coordinated regulation on fungal cells, with early induction (12–24 h) of lipoxygenase (*D. nLOX-1/2/3*) and peroxidase (*D. nPOD-1/2/3*) genes triggering oxidative burst and lipid peroxidation (Figure 9A,B). Concurrent disruption of phospholipid metabolism resulted in irreversible membrane damage within 24 h (Figure 8). Transcriptome analysis further confirmed the dual antifungal strategy of A4, showing upregulation of ergosterol synthesis (*ERG11*), fatty acid desaturation (*OLE1*), and lipoxygenase genes (*LOX1-4*) alongside apoptosis-related genes (*NUC1*, *AIF1*, *Cas7*) as early as 12 h post-treatment (Figure 7C). Notably, the strain activated a conserved apoptotic pathway, with significant upregulation of caspase genes *D. nCas6*, *D. nCas7*, and *D. nCas9* by 5.62-, 3.13-, and 2.59-fold at 24–36 h, respectively (Figure 9C), ultimately leading to organelle degradation. This cascade progressing from membrane damage to caspase-mediated apoptosis parallels reported *Bacillus* antifungal mechanisms [19], highlighting the strain’s unique capacity for multi-pathway interference and apoptosis induction.

## 5. Conclusions

*Bacillus velezensis* A4 exhibits broad-spectrum antifungal activity through a multi-target synergistic mechanism. Its cell-free supernatant (CFS), containing lipopeptides and antifungal proteins as key antimicrobial components, effectively suppresses the growth of pathogenic fungi in vitro and in vivo. Integrated transcriptomic and metabolomic analyses further demonstrate that the CFS temporally coordinates a sequential response involving oxidative stress, membrane damage, and caspase-mediated apoptosis, collectively leading to fungal inhibition. This study systematically elucidates the biocontrol mechanism of strain A4, providing a theoretical foundation for advancing sustainable, microbially based alternatives to chemical pesticides.

## Data Availability

The original contributions presented in this study are included in the article/Supplementary Materials. Further inquiries can be directed to the corresponding author.

## Supplementary Materials

The following supporting information can be downloaded at https://www.mdpi.com/article/10.3390/jof11120851/s1. Table S1. *D. nobilis* membrane oxidation qPCR primer sequences; Figure S1. Effect of CFS on mycelial morphology of pathogenic fungi; Figure S2. Linoleic acid metabolic pathway; Figure S3. The inhibitory effect of crude extracts on kiwifruit soft rot and the diameter of pathogens.
