# Supplementary material for: Bacillus velezensis A4 Against Fungal Pathogens via Membrane Integrity Disruption and Cellular Dysfunction in Fungal Pathogens
Source: J Fungi (Basel). 2025 Nov 29;11(12):851. doi: 10.3390/jof11120851 (PMC12733517; doi:10.3390/jof11120851)
Supplement: Supplementary file 1 [file jof-11-00851-s001.zip › jof-3986516-supplementary.pdf]

Tab. S1 *D. nobilis* membrane oxidation qPCR primer sequences

Gene number	Primer name	Primer sequence (5′→3′)
EVM0009020	*D. nLOX-1*-F	CAAACGCAGCTGGAAAGTTC
	D. nLOX-1-R	TGGGTGTAGTTGGTGAGGA
EVM0015302	*D. nLOX-2*-F	CCGCTATCCACCCCATTTG
	D. nLOX-2-R	CGTGAAGTCCTTGATCGAGTC
EVM0010774	*D. nLOX-3*-F	GTGTTCCTGTGCATGTTTAACC
	D. nLOX-3-R	GGTCCTCGTCATATTTTGCCC
EVM0002653	*D. nPOD-1*-F	GACAACTCATCCTTCAACTTGG
	D. nPOD-1-R	CGAAGTAGATGTCCGCACG
EVM0012053	*D. nPOD-2*-F	TTATAAGCGTCCTGGTCATGG
	D. nPOD-2-R	GTCATAGGAAATGTTGCGGC
EVM0013784	*D. nPOD-3*-F	GCGAGCACAGCATAAACAAG
	D. nPOD-3-R	AGACACCATCCAAGAAACCG
EVM0013010	*D. nCas7*-F:	GATGTTATCCTGTGGAGCGG
	D. nCas7-R:	GTCTGGTTTTGGTTCTGCTTG
EVM0003630	*D. nCas9*-F:	GCTGTATTTTGGTTGCGTCTG
	D. nCas9-R:	TCCTTGGTTTCCTATTCTGGC
EVM0006724	*D. nCas6*-F:	GTACAAAACCCGAAGCTGTTC
	D. nCas6-R:	GTGTATCTCCCCTTGGTCAATG

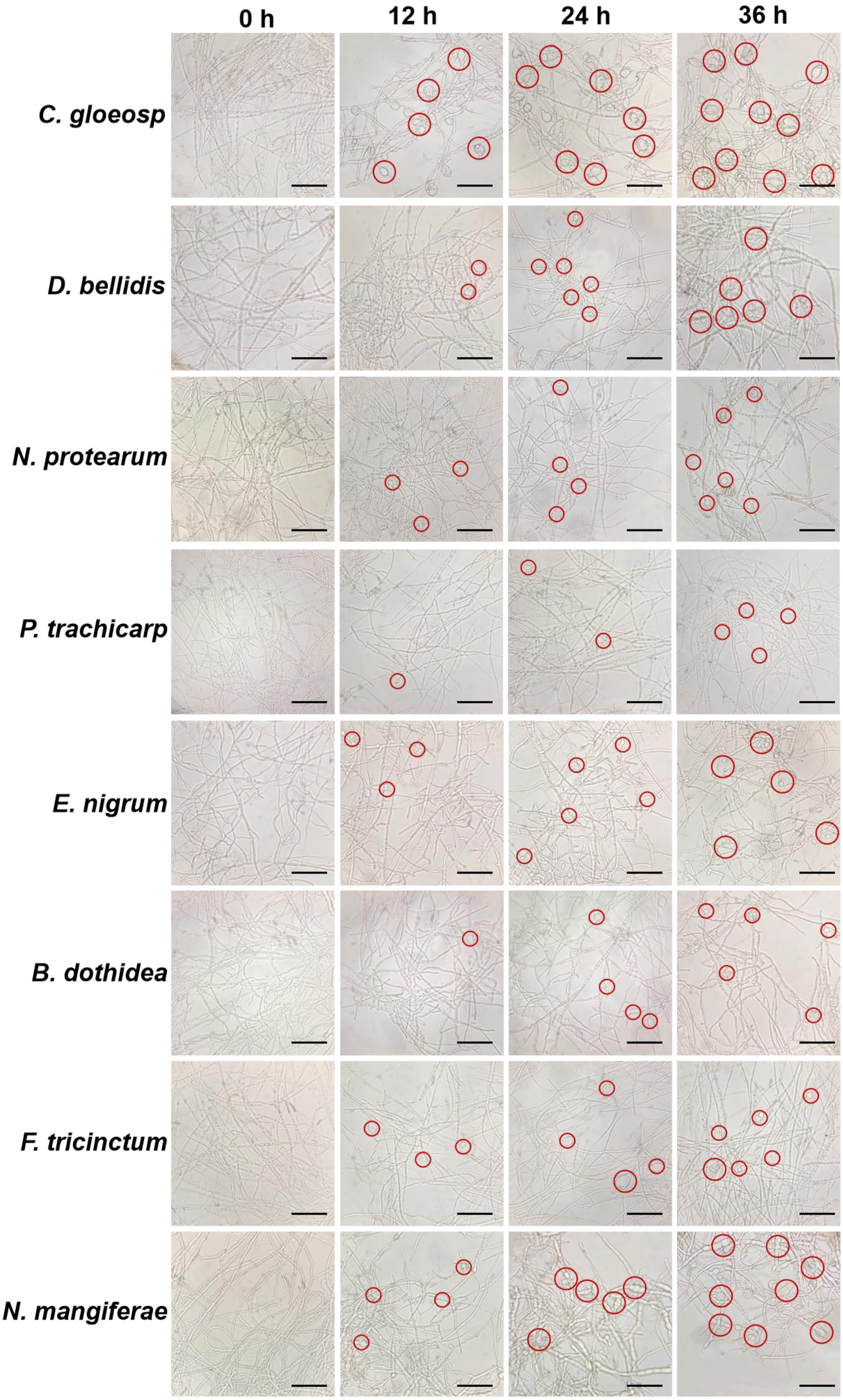

Fig. S1 Effect of CFS on mycelial morphology of pathogenic fungi

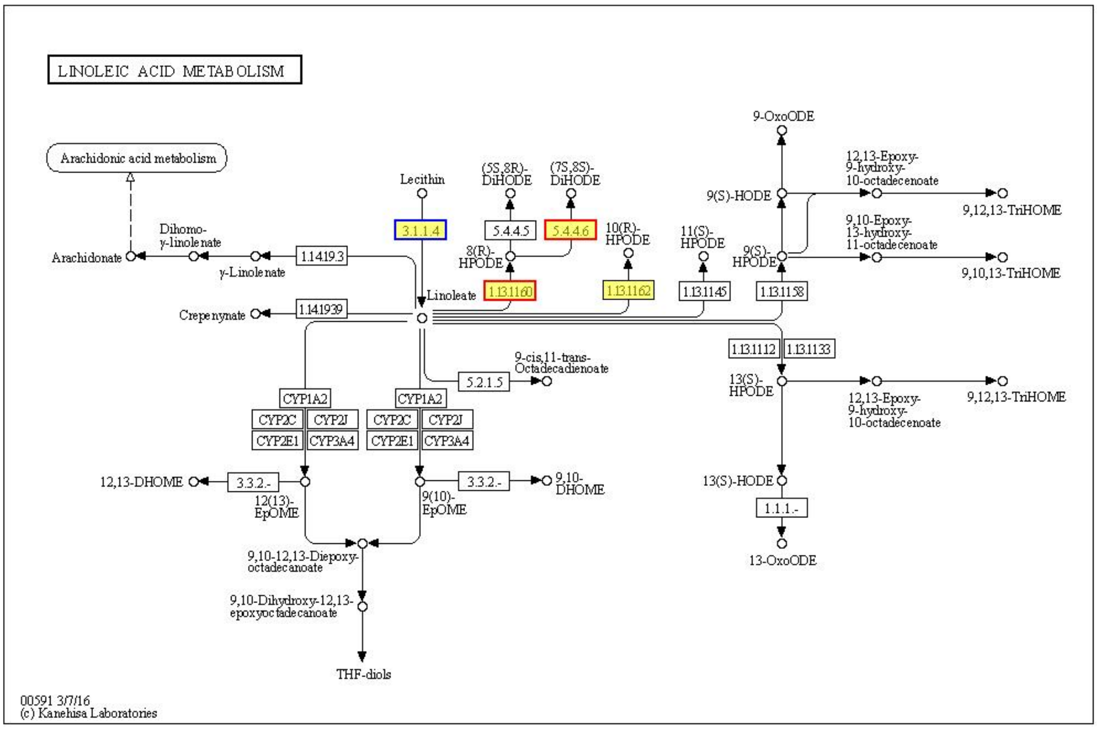

Fig. S2 Linoleic acid metabolic pathway

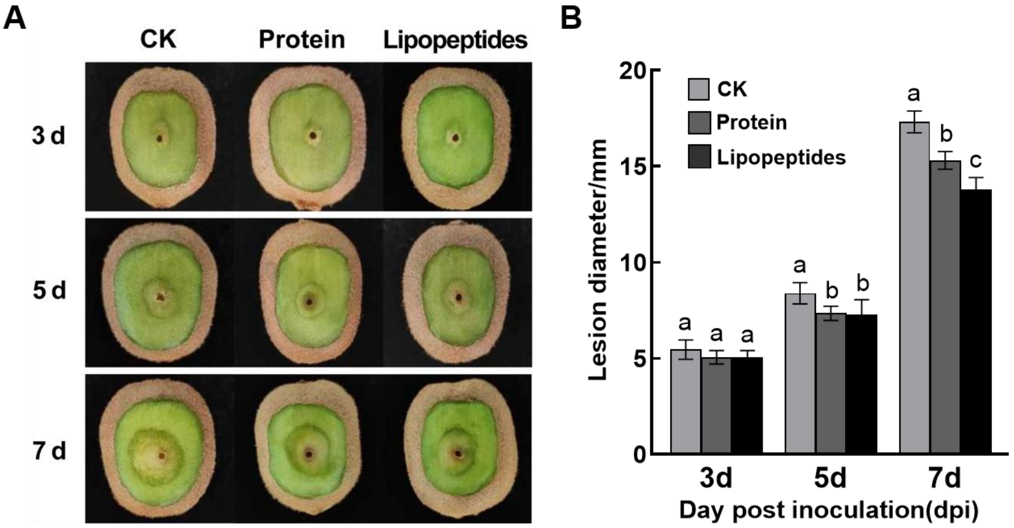

Fig. S3. The inhibitory effect of crude extracts on kiwifruit soft rot and the diameter of pathogens. (**A**) Photographs of diseased fruits were taken 3, 5, and 7 days after treatment. (**B**) Quantification of lesion diameters. Histograms with different letters indicate significant differences according to Duncan's multiple range tests at $p < 0.05$.
